# Lessons learned from the search for genes responsible for rare Mendelian disorders

**DOI:** 10.1002/mgg3.233

**Published:** 2016-07-18

**Authors:** Nara L. Sobreira, David Valle

**Affiliations:** ^1^ McKusick‐Nathans Institute of Genetic MedicineJohns Hopkins University School of MedicineBaltimoreMaryland 21205; ^2^ Department of PediatricsJohns Hopkins University School of MedicineBaltimoreMaryland 21205

The last decade has witnessed dramatic improvements in DNA sequencing technology with reduced cost, increased throughput, and improved analytic tools and resources. A consequence of this technologic revolution is the rapid emergence of approaches applying these next‐generation sequencing (NGS) methods to many areas of medicine including discovery research and clinical diagnosis. Some consequences of this revolution include the ability to make molecular diagnosis for thousands of inherited phenotypes; molecular characterization of cancers that enable diagnostic refinement and individualized therapy; elucidation of pharmacogenetic susceptibilities, and enumeration of individual architectures of genetic variation conferring risk for common complex traits such as coronary artery disease, diabetes, and neuropsychiatric disease. These newly acquired capabilities form the cornerstone for individualized or precision medicine of the future.

## Current Methods

These methods can be considered by the target to be sequenced: (1) a specific disease gene, for example, *BRCA1*; (2) a “panel” or set of genes responsible for a phenotype with locus heterogeneity, for example, ~31 genes that can cause familial hypertrophic cardiomyopathy; (3) whole‐exome sequencing (WES) that targets ~1.5% of the genome containing the coding exons of all of ~20,000 protein coding genes in our genome; and (4) whole‐genome sequencing (WGS) that targets our entire genome. In the clinical setting, there is much more experience with the first three methods; each has strengths and weaknesses but when used appropriately, each has great clinical utility. In the disease gene discovery efforts for rare Mendelian disorders, there has now been considerable experience with WES. One key difference between the clinical and research applications is that, in the latter, it is often possible to sequence several members of a pedigree and evaluate candidate variants using segregation and other genetic models. Moreover, WES is particularly suited for the search of genes responsible for rare Mendelian disorders because our ability to analyze and interpret variants in this segment of the genome is far better than in nonprotein coding segments. Using these approaches, more than a thousand disease genes have been identified (Chong et al. 2015). In what follows, we will focus on WES and what has been learned from its application in the clinic to diagnose rare disorders and its use in research in the quest to identify genes responsible for rare Mendelian disease.

## Some Lessons Learned

Since the time of Mendel (Opitz and Bianchi 2015), genetics has explored the relationship of genotype to phenotype. While initial studies uncovered rather direct connections (e.g., homozygosity for recessive loss‐of‐function alleles leads to deficiency of an enzyme and the downstream metabolic and clinical abnormalities), we are increasingly discovering more complicated models.

### One gene/many phenotypes

As of 8 June 2016, OMIM describes 4739 phenotypes with known molecular basis and 3564 genes with causative variants (~1.3 discrete phenotypes per disease gene) with some genes (e.g., LMNA, COL2A1, FGFR3) causing more than 10 unrelated and/or partially overlapping phenotypes. As these numbers increase, it will be interesting to look for biological differences in those genes in which variation can produce many as compared to those that produce only one phenotype.

### One phenotype/many genes

Locus heterogeneity has been known for some time (e.g., Noonan syndrome, retinitis pigmentosa, and dilated cardiomyopathy), but the frequency of overlap in phenotypic consequences of pathogenic variants in multiple genes is emphasized by the ongoing gene discovery studies. OMIM lists examples of locus heterogeneity as phenotypic series, and as of 8 June 2016, 365 are described (Amberger et al. 2015). In many instances, overlapping phenotypes result from pathogenic variants in the genes encoding proteins, that all function in a particular biologic system (e.g., genes encoding components in the cardiac contractile apparatus and cardiomyopathy). In other instances, exploration of locus heterogeneity leads to identification of previously unappreciated interactions between apparently discrete biological systems (Goh et al. 2007; Vidal et al. 2011). Appreciation of the origins of these phenotypic overlaps promises to improve our understanding of human disease.

### One proband/blended phenotypes/multiple genes

Recent studies primarily using WES to study patients with unrecognizable phenotypes have identified individuals affected by more than one rare Mendelian disease. The resulting “blended phenotype” defies diagnosis and undermines the clinical dogma that we should seek a single explanation for a complex phenotype. For example, Yang et al. (2014) reported that, of 504 patients with a molecular diagnosis, 23 (4.6%) had blended phenotypes resulting from two single‐gene defects and Retterer et al. (2015) reported analysis of 3040 probands, identifying 25 that had two genetic diagnoses and three with three distinct genetic diagnoses.

### Phenotypic expansion

Virtually no clinical phenotypes are identical in all affected individuals. Thus, careful study of many affected individuals is necessary to define the full phenotypic spectrum of a particular disease. A corollary is that, for rare disorders, the number of individuals described is often quite small and we underestimate the full extent of the clinical phenotype. Thus, the search for genes responsible for rare Mendelian disorders often identifies a known disease gene in an individual whose phenotype differed in some significant way from that of previously described affected individuals. Recognition of this “phenotypic expansion” greatly improves our understanding of the phenotypic consequences of variation in a known disease gene. A review of results of the first 3 years of the Centers for Mendelian Genomics identified phenotypic expansion associated with 198 of 956 disease gene discoveries (Chong et al. 2015). Interestingly, apparent phenotypic expansion may also reflect effects of a modifier locus. Understanding of this mechanism often suggests alternative therapeutic approaches (Corvol et al. 2015). Moreover, the differentiation between phenotypic expansion and blended phenotype is not always clear. Thus, in the future, what we thought was a phenotypic expansion may, in some instances, be reclassified as blended phenotype and vice versa.

## The Problem of Unsolved Cases

Despite these advances, in more than half of the individuals with a rare Mendelian phenotype who undergo a clinical or research WES, the responsible gene and causal variants cannot be identified (Yang et al. 2014; Chong et al. 2015; Retterer et al. (2015). Some reasons for this relatively low yield include unappreciated phenotypic heterogeneity; locus heterogeneity; somatic and germline mosaicism; missense variants of uncertain functional significance; variants difficult to detect by WES including indels, CNVs, or chromosomal rearrangements; incorrect mode of inheritance investigated; causative coding variants not sequenced by the WES; causative variants in regulatory region; and inadequate communication between clinicians and basic scientists with knowledge of particular genes, proteins, or biological systems. To address this lack of sensitivity, a variety of strategies can be considered to improve and complement the analysis of the WES data.

### Detailed phenotyping

Comprehensive phenotyping of the individuals being sequenced is a vital step in the disease gene identification process. This information is critical for identifying unrecognized phenotypic and locus heterogeneity as well as increasing suspicion for phenotypic expansion and blended phenotypes. Tools such as PhenoDB (Sobreira et al. 2015) facilitate accumulation of standardized and searchable phenotypic features, the description of the individual(s) being investigated, and the integration of this information into the analysis pipeline for either WES and/or WGS.

### Reanalysis of WES data with methods that facilitate identification of indels and CNVs

A variety of approaches for detection of indels and CNVs have been developed that have identified causative variants in novel Mendelian genes that were previously overlooked. For example, Lalani et al. (2016) identified CNVs in *TANGO2* as the cause of recurrent metabolic encephalomyopathic crises associated with rhabdomyolysis, cardiac arrhythmias, and neurodegeneration (OMIM616878).

### Investigation of unusual modes of inheritance

Analysis pipelines often consider only standard autosomal dominant, recessive, and X‐linked modes of inheritance. Less standard modes of inheritances such as autosomal dominant with incomplete penetrance, maternal and paternal imprinting, sex‐limited phenotypes, Y‐linked inheritance, or X‐linked genes in the pseudoautosomal regions or in genes that escape X inactivation are not considered. Incorporating these models in the analysis pipeline can lead to successful identification of the causative variants and genes. For example, using an analysis strategy that incorporates knowledge of imprinted genes, Chacón‐Camacho et al. (2016) identified a rare variant in the maternally imprinted *ZDBF2* as a strong candidate gene for the palpebral coloboma‐lipoma syndrome (MIM167730).

### Whole‐genome sequencing (WGS)

WGS has been used to solve rare cases of Mendelian phenotypes. Sobreira et al. (2010) used WGS together with linkage analysis to identify loss‐of‐function variants in *PTPN11* as the cause of metachondromatosis. However, WGS is still ~3 times more expensive and much more difficult to analyze than WES because of the difficulty in interpreting the functional consequences of variants in noncoding sequence. However, WES also has significant limitations. Preparation of WES sequencing libraries involves a selection step to enrich for the exome that typically involves hybridization with RNA baits complementary to exon sequences (http://www.agilent.com/cs/library/usermanuals/Public/G7550-90000.pdf). This hybridization step is nonlinear and incomplete, capturing 85–95% of target sequences. Thus, WES often fails to detect CNVs and may not sequence all intended exons. For example, Belkadi et al. (2015) estimated that ~3% of coding variants missed by WES were detected by WGS. Thus, WGS has higher sensitivity for certain coding variants, indels, CNVs, chromosomal rearrangements, or causative variants in regulatory region. For example, Herdewyn et al. (2012) identified (GGGGCC)n repeat expansions in C9orf72 as a cause of familial amyotrophic lateral sclerosis using WGS and Goos et al. (2016) identified intragenic exon deletions (of sizes 84.9, 8.6, and 5.4 kb) in the TCF12 gene in three different families with coronal synostosis using WGS. Additionally, WGS in combination with linkage analysis, homozygosity mapping, or RNAseq facilitates the identification of noncoding or pathogenic splicing variants. The value of RNAseq data to evaluate the functional significance of noncoding variants is emphasized by the identification of a noncoding splicing variant in a novel disease gene causing muscular dystrophy (Gonorazky et al. 2015).

### Somatic mosaicism investigation

Sequencing of affected and unaffected tissue from the same individual using either WES or WGS has solved both cancer and noncancer phenotypes resulting from somatic mosaicism. Typically, this involves deeper than usual sequencing, together with an analysis strategy that identifies variants that are found at a lower frequency than that expected for heterozygous germline variants. For example, using WES of DNA isolated from affected and unaffected tissue, Lindhurst et al. (2011) identified a somatic activating variant in the AKT1 gene as the cause of Proteus syndrome. Subsequently, somatic mosaicism has been shown to be the cause of several other phenotypes including congenital hemangiomas (OMIM163000), X‐linked acrogigantism syndrome (OMIM300942), Sturge–Weber syndrome (OMIM185300), and many others (Shirley et al. 2013; Ayturk et al. 2016; Daly et al. 2016).

## The Value of Data Sharing

Regardless of the sequencing strategy, the endgame for disease gene identification often comes down to identifying multiple affected individuals with similar phenotype and candidate variants in the same gene and/or evaluating the functional consequences of candidate variants in a few candidate genes. This process can be greatly facilitated by connecting with other clinicians with patients with variants in the same candidate gene and with basic scientists with special expertise and/or model organisms with defects in the orthologous genes. GeneMatcher (www.genematcher.org) is a freely accessible website that facilitates such data sharing. The site allows investigators to post a gene(s) (by gene symbol, base pair position, Entrez‐ or Ensembl‐Gene ID) of interest automatically sending reciprocal emails to investigators who post the same gene. Subsequent follow‐up is at the discretion of the submitters. Only submitters have access to their own entry data and may edit them or delete them at will. There is also an option to match, based upon OMIM^®^ number, genomic location, and, as of October 2015, on phenotypic features. If a match is not identified at the time of submission, the gene(s) of interest continues to be queried as new entries are submitted. As part of the Matchmaker Exchange (MME) (Philippakis et al. 2015), GeneMatcher has also developed an application programming interface (API, available upon request) that was implemented in August 2015 and allows the GeneMatcher users to submit their data to query PhenomeCentral (https://phenomecentral.org/) and/or DECIPHER (https://decipher.sanger.ac.uk/). The user has the option of querying one or both databases by gene names, genomic location, OMIM^®^ number, and/or phenotype information; the match is carried out automatically with submitters receiving simultaneous email notification, and follow‐up is at the discretion of the submitters. As of 1 June 2016, 4706 genes were submitted to GeneMatcher by 1810 individuals from 55 countries. There have been 6147 matches involving 1339 genes (123 matches with PhenomeCentral and 127 with DECIPHER) that have enabled collaborations and the description of novel Mendelian phenotypes and novel Mendelian disease genes, such as SPATA5, HNRNPK, TELO2, RSPRY1, HIVEP2, CHAMP1, and others (Au et al. 2015; Faden et al. 2015; Hempel et al. 2015; Tanaka et al. 2015; Steinfeld et al. 2016; You et al. 2016).

## Prospects for the Future

As of 8 June 2016, only 3564 of ~20,000 human protein coding genes have been found to have a phenotype‐causing variant (~16.2%), the molecular basis of at least 3425 phenotypes is not known, and many more Mendelian phenotypes have not yet been described. These data together with the fact that the vast majority of the disease‐causing variants investigated up to now are single‐nucleotide variants in the coding region show us that there is a long way to go if we are to identify the molecular basis of every Mendelian phenotype, the details of the phenotypes, and the origins of the associated phenotypic variability. The development of novel genomic and functional laboratory methods, the improvement of known approaches, better phenotyping and the sharing of the data including better partnership with the patients will all be fundamental to the understanding of the diseases mechanisms and development of treatments.

## Conflict of Interest

None declared.
